# Conserved +1 translational frameshifting in the *Saccharomyces cerevisiae* gene encoding YPL034W

**DOI:** 10.1016/j.jbc.2025.110891

**Published:** 2025-11-04

**Authors:** Ivaylo P. Ivanov, Swati Gaikwad, Byung-Sik Shin, Alan G. Hinnebusch, Thomas E. Dever

**Affiliations:** Division of Molecular and Cellular Biology, Eunice Kennedy Shriver National Institute of Child Health and Human Development, National Institutes of Health, Bethesda, Maryland, USA

**Keywords:** protein synthesis, translation control, ribosome, RNA, Transfer RNA (tRNA), frameshift, recoding

## Abstract

Cells have developed exquisite mechanisms to ensure accurate translation of mRNA, including preventing a change in reading frame during translation elongation. A minority of chromosomally encoded genes have evolved sequences that subvert standard decoding to program +1 translational frameshifting, either constitutively or in response to external stimuli. In the yeast *Saccharomyces cerevisiae*, three chromosomal genes are known to employ programmed +1 translational frameshifting for expression of full-length functional products. Here, we identify a fourth yeast gene, *YFS1*, encompassing the existing predicted open reading frame *YPL034W*, with conserved programmed +1 frameshifting. Like the previously known examples, *YFS1* appears to exploit peculiarities in tRNA abundance in *S. cerevisiae* to promote frameshifting.

As determined more than half a century ago, translation begins with ribosomes initiating at a specific start codon, usually AUG, which commits them to translating one of the three possible reading frames of an mRNA (1). Elongation then proceeds by decoding adjacent triplet nucleotides until one of three stop codons is encountered (2). Loss of frame maintenance has severe consequences. Loss of protein expression can lead to haploinsufficiency (3), while translation of aberrant peptides that terminate prematurely or with C-terminal extensions can lead to dominant negative genetic consequences and activation of cellular stress responses (4, 5, 6, 7). To avoid these deleterious consequences, cells invest considerable energy and resources to maintain the translation reading frame after ribosomes have committed to the start of translation. Nevertheless, a small group of genes have evolved sequences that act in *cis* to subvert standard decoding, often for regulatory purposes—a process known as recoding (8). Recoding encompasses phenomena like stop codon readthrough, ribosome hopping, and ribosomal frameshifting. In the latter, a fraction of translocating ribosomes slip forward (3′) or backward (5′) resulting in “+” or “-” frameshifting (9, 10). Most known cases of programmed frameshifting involve either +1 or −1 frameshifts that, while superficially similar, are mechanistically distinct. Well studied examples of −1 frameshifting result from simultaneous slippage of tRNAs relative to mRNA when both the A and P sites of the ribosome are occupied by tRNAs decoding a heptanucleotide sequence with the signature motif X-XXY-YYZ (11), wherein the tRNA decoding the triplet XXY can also decode XXX and the tRNA decoding YYZ can also decode YYY. By contrast, +1 frameshifting usually occurs when the ribosomal P site is occupied by peptidyl-tRNA, but the A site is empty owing to slow decoding of the A site triplet (12, 13). In addition to a defined frameshift site sequence, most cases of programmed frameshifting include additional sequences, present 5′ or 3′ of the shift site, that dramatically alter the efficiency of frameshifting (9).

Work on translation of the gag-pol fusion proteins encoded by the Ty1 and Ty3 retrotransposons of *Saccharomyces cerevisiae* provided important insights into the mechanism of +1 frameshifting. Ty1 frameshifting occurs on the heptanucleotide sequence CUU-AGG-C when the CUU Leu codon is in the ribosome P site and AGG Arg codon is in the A site (14). This sequence alone, without any additional *cis*-elements, supports ≥40% frameshifting depending on the reporter. A larger cassette, including additional sequences naturally surrounding the shift site, yields somewhat lower +1 frameshifting of 12 to 20%, suggesting the potential presence of frameshift suppressing sequences (15, 16). The CUU-AGG-C sequence also directs +1 frameshifting in *ABP140* (17), one of three *S. cerevisiae* chromosomal genes known to employ programmed +1 frameshifting. Another yeast gene requiring +1 frameshifting for expression, *EST3*, employs a derivative of the Ty1 shift site, CUU-AGU-U (18). The third *S. cerevisiae* chromosomal gene, *OAZ1*, has the shift site GCG-UGA-C (19), which shares the same P-site codon as the Ty3 shift site, GCG-AGU-U (20). The latter, in turn, shares its A-site triplet with that of the *EST3* shift site. If the shift prone Ty1 sequence CUU-AGG-C is present in-frame within a main Open Reading Frame (mORF), which otherwise does not require +1 frameshifting, it is predicted to trigger a high frequency of spurious switches in the reading frame, producing truncated polypeptides, which could be detrimental to cell physiology. Consistent with this prediction, computational analysis revealed a significant underrepresentation of the heptanucleotide CUU-AGG-C in-frame in yeast mORFs, even when codon usage and nucleotide composition are considered (21).

Here, we show that the previously annotated yeast ORF *YPL034W* is part of a larger conserved ORF lacking an initiation codon that is instead accessed following initiation at an overlapping ORF initiated further upstream. A fraction of ribosomes initiates on the first ORF then frameshifts in the +1 direction while translating the heptanucleotide sequence CUU-AGG-C. This frameshift sequence, and the frameshifting itself, is conserved in most sequenced members of the budding yeast *Saccharomycetaceae* family. We propose to name the newly identified gene Yeast Frame Shift 1 (*YFS1*).

## Results

### Evidence that expression of *YPL034W* requires +1 frameshifting

**Figure 1:**
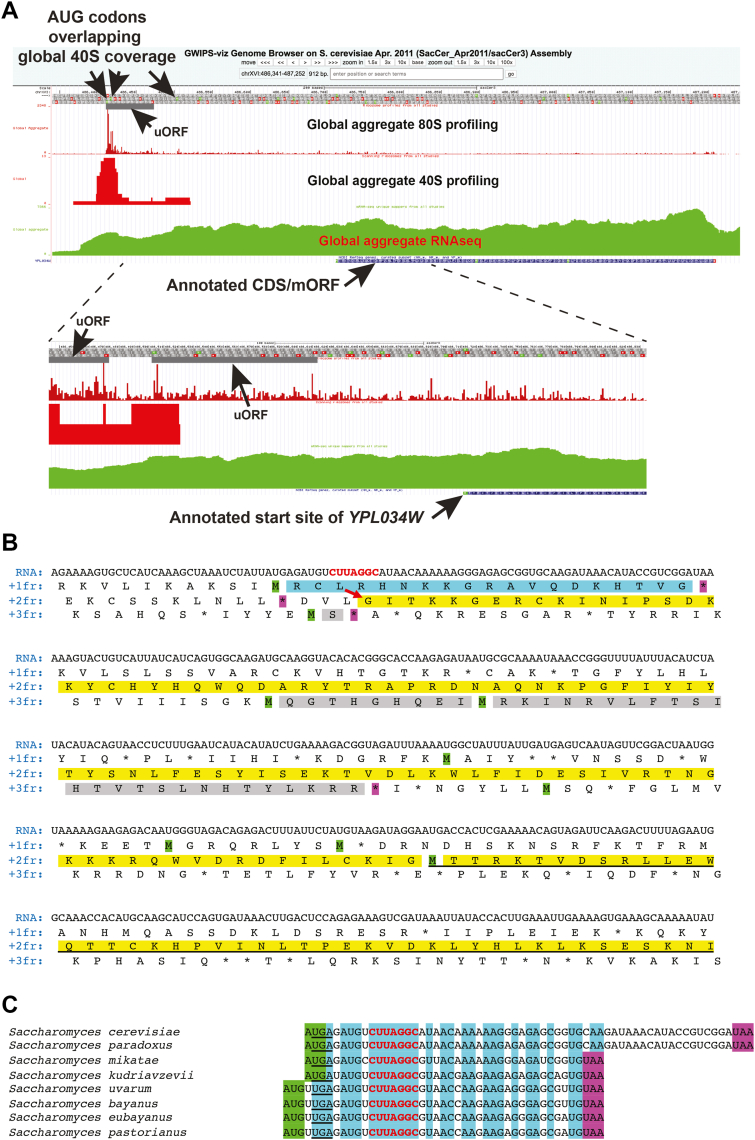
**Aggregate ribosome profiling and the structure of the YPL034W mRNA provides evidence for ribosome frameshifting**. *A*, screen shot from the GWIPS-vis browser (https://gwips.ucc.ie/) of global aggregate 80S and 40S ribosome profiling and RNAseq coverage in the vicinity of *YPL034W*. The amino acid sequence in all three reading frames of the positive strand is shown as a band near the top (the exact sequence can be seen in (*B*)). AUG start codons are in *green*, and stop codons are in *red*. The position of the first uORF (*gray bar*) is indicated; and the global aggregate 80S, global aggregate 40S, and RNAseq coverage are displayed below the amino acid sequence. The positions of AUG codons corresponding to global aggregate 40S coverage are indicated by downward arrows on the amino acid sequence. The position and sequence of the annotated *YPL034W* ORF is shown below the RNA coverage. *Lower panels*: Blow up of the region spanning the first uORF to the annotated mORF, with the position of a second putative uORF also indicated. *B*, nucleotide and amino acid sequence of approximately the first two-thirds of *S. cerevisiae YFS1* mRNA. The sequence begins with the transcription start site as determined by CAGE data (57). The mRNA nucleotide sequence is on top, and the simulated translated amino acid sequence in all three frames is shown below with the different frames indicated on the *left*. Methionine residues, encoded by putative AUG initiation codons, 5′ of the annotated AUG start codon of *YPL034W*, are highlighted in *green*. Relevant in-frame stop codons are in *magenta*. The sequence of the first uORF of *YPL034W* (ORF1 of *YFS1*) is highlighted in *cyan*. The proposed encoded peptide of ORF2 of *YFS1* is highlighted in *yellow*; the part constituting the previously annotated *YPL034W* is underlined. Two uORFs/iORFs discussed in the main text are highlighted in *gray*. The putative frameshift site of *YFS1* in ORF1 is shown in *red* letters, and the position and direction of the putative +1 translational frameshift is indicated by a *red arrow*. *C*, nucleotide sequence alignment of ORF1 (annotated uORF) of *YFS1.* The sequences of ORF1 from 8 species belonging to the *Saccharomyces* genus are aligned on the *right*. Species names are on the *left*. Start codons are highlighted in *green*. Stop codons are highlighted in *magenta*. Nucleotides conserved in all 8 species are highlighted in *cyan*. Nucleotides constituting the putative frameshift site are in *red*. The in-frame UGA stop codon bracketing the 5′ end of ORF2 in the +1 frame is underlined.

During a search for conserved upstream open reading frames (uORFs) in yeast, we visually examined the mRNA of *YPL034W* for its translation potential using global aggregate ribosome profiling data available at GWIPS-viz (22). Aggregate 80S ribosome profiling coverage was consistent with translation initiating at an AUG codon at positions 486,411 to 486,413 of chrXVI (SacCer_Apr2011/sacCer3 assembly) located 298 nucleotides (nt) upstream of the annotated AUG start codon of *YPL034W* (at positions 486,712-486,714) indicated at SGD (https://www.yeastgenome.org). Ribosome footprints proximal to this upstream AUG codon were denser than the footprints in the annotated mORF of *YPL034W* (Fig. 1*A*), which led us initially to consider the AUG as the start codon of a short uORF of 20 codons. Surprisingly, however, there was no break in 80S ribosome coverage between the stop codon of the suspected uORF and the annotated mORF beginning 239 nt downstream. This was true even after taking into account the presence of a potential additional uORF of 111 nt starting 30 nt downstream of the first suspected uORF (Fig. 1, *A* and *B*). The lack of any change in ribosome coverage in the region before and after the annotated AUG start codon of *YPL034W* suggested the possibility that this region is translated without interruption starting somewhere within the suspected first short uORF.

Performing 40S ribosome profiling Archer *et al.* previously demonstrated accumulation of 40S footprints at annotated initiation codons and depletion of 40S footprints immediately downstream (23). The GWIPS-viz profile of this 40S profiling data were consistent with initiation at the AUG start codon of the suspected first uORF and possibly at the AUG start codon of the second potential uORF beginning 30 nt further downstream (Fig. 1, *A* and *B*). The 40S data did not, however, provide support for initiation at the annotated AUG start codon of the mORF. Mapping of transcription start sites (TSSs) by Cap Analysis of Gene Expression (CAGE) revealed that the major cluster of TSSs found at *YPL034W* maps ∼37 nt upstream of the AUG start codon of the potential first uORF (http://yeastss.org/), making the latter the first AUG encountered in the mRNA reading downstream from the TSS. Together, these data support the hypothesis that the AUG start codon of the potential uORF1 is actually the primary start codon of *YPL034W.*

**Figure 2:**
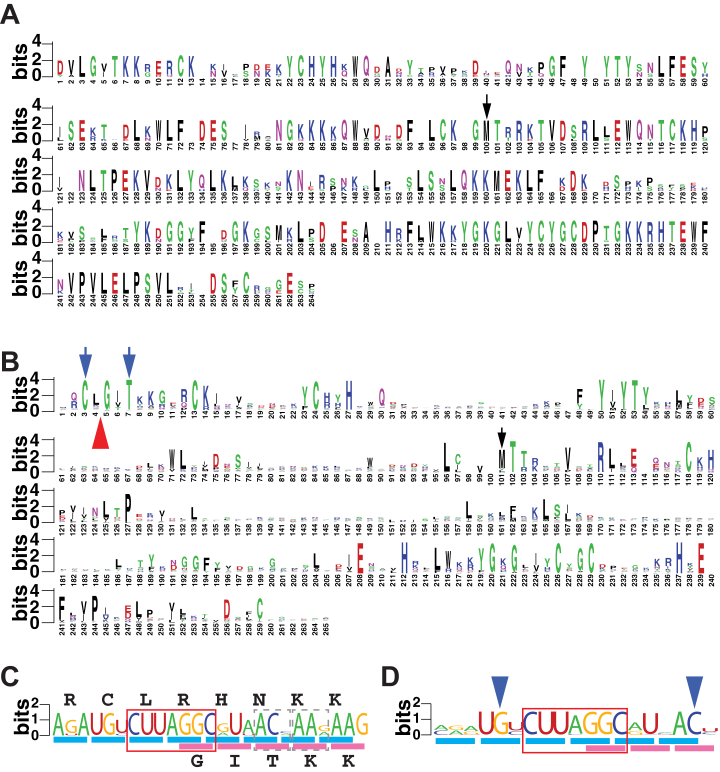
**Conservation of the amino acid sequence of Yfs1 upstream of the annotated YPL034W and of the nucleotide sequence of the frameshift site.***A* and *B*, Weblogo representation of the amino acid sequence conservation based on (*A*) alignment of ORF sequences (upstream in-frame stop codon to annotated stop codon of YPL034W) from 8 species belonging to the genus *Saccharomyces* or (*B*) alignment of simulated translations of sequences from 81 species belonging to the fungal order *Saccharomycetales* – 52 with and 29 without a frameshifting site. Insertions relative to the *S. cerevisiae* sequence were removed, and therefore, numbering corresponds to the *S. cerevisiae* sequence. In both (*A* and *B*) the position of the currently annotated AUG (YPL034W) start codon is indicated by a *black arrow*. In (*B*) the position of the frameshift site is indicated by a *red arrow*; the position of the two conserved codons flanking the frameshift site (shown in *D*) are indicated by *blue arrows*. *C* and *D*, Weblogo representation of the nucleotide sequence conservation flanking the frameshift site of *YFS1* based on (*C*) sequences from 8 species belonging to the genus *Saccharomyces* or based on (*D*) the same sequences from the 52 species with frameshift sites belonging to the fungal order *Saccharomycetales* indicated in (*B*). Triplets in the “0” frame are underlined with *blue bars*, while those in the “+1” frame are underlined in magenta. The CUU-AGG-C Ty1-like shift site is boxed in *red*. In (*C*) two triplets showing synonymous substitution pattern in the +1 frame following the frameshift site are boxed with *dashed lines*. The amino acid sequence in the 0 and + 1 frame in *S. cerevisiae* are shown above and below the conservation logo, respectively. In (*D*) triplets (UGY Cys and ACY Thr) showing synonymous substitution patterns before and after the shift site, in the “0” and “+1” frame, respectively, are indicated by *blue arrows*.

The sequence of the first 465 nt of the *S. cerevisiae YPL034W* encoding mRNA and the features we identified within it are shown in Figure 1*B*. The coding region corresponding to the annotated CDS (coding sequence, here also referred to as the mORF) in *S. cerevisiae* extended upstream without intervening in-frame stop codons to just 3′ of the start codon of the first uORF, which is in a different frame (Fig. 1*B*). Sequences from 7 additional homologs of *YPL034W* from yeast belonging to the *Saccharomyces* genus were obtained for analysis. In all 7 sequences, the bracketing (upstream) in-frame stop codon for the mORF (underlined in Fig. 1*C*) was again found just after the start codon of the first uORF, which is also conserved in all 8 *Saccharomyces* species. No available in-frame AUG codons exist to initiate translation of this extension of the mORF, as is also true for all analyzed *Saccharomyces* orthologs. For all 8 sequences, the reading frame corresponding to the annotated mORF was conceptually translated from the first in-frame stop upstream of the annotated start codon to the next in-frame stop codon (the annotated stop codon of the mORF). The sequences were then aligned using ClustalX and displayed as a logogram (Fig. 2*A*). The alignment showed that 44 of 96 amino acid residues 5′ of the annotated AUG start codon are conserved in all 8 species, consistent with the idea that this region is translated and that its product has biological function. We considered the possibility that the conserved extension is initiated by a near-cognate start codon, as has been reported for various genes in both in *S. cerevisiae* and in other species (24, 25, 26, 27, 28). However, no conserved near-cognate start codon was apparent 3′ of the upstream in-frame stop codon and 5′ of the first absolutely conserved amino acid residue, T6, seemingly precluding the possibility of near-cognate initiation of the extension.

We next focused our attention on the first potential uORF. In all 8 species, this uORF overlaps the putative N-terminal extension of the *YPL034W* mORF in the +1 frame. However, even in these relatively closely related species, neither the position of its start nor its stop codon was invariant (Fig. 1*C*). Aligning the nucleotide sequences of the predicted first uORF for the eight *Saccharomyces* homologs of *YPL034W* (Fig. 1*C*) immediately suggested a mechanism for the translation of the putative N-terminal extension. Seven absolutely conserved nucleotides, CUU-AGG-C, were found within the potential uORF. Of note, the CUU triplet is in-frame with the uORF AUG start codon; moreover, in the +1 reading frame of the motif (the frame of the mORF) there are no stop codons between the motif and the annotated mORF start codon (Fig. 1*B*). Strikingly, this conserved sequence is identical to the known +1 frameshift site required for translation of gag-pol in the retrotransposon Ty1 and found in the yeast chromosomal gene *ABP140*. As noted above, a variant of this sequence, CUU-AGU-U, is responsible for the +1 frameshifting in the yeast chromosomal gene *EST3*. Furthermore, almost immediately following the motif (putative shift site) in *YPL034W*, the pattern of nucleotide conservation is consistent with synonymous substitutions in the +1 frame, *i.e.* triplets ACN (Thr) and AAR (Lys) (Fig. 2*C* - boxed in grey). Together, these findings strongly suggested that translation of the mORF of *YPL034W* proceeds by initiation at the AUG start codon of the first potential uORF and then shifts into the continuous reading frame of the mORF owing to slow decoding of the AGG triplet with subsequent decoding of the GGC triplet instead following a +1 frameshift. Henceforth, to distinguish between the newly identified gene product and the previously annotated (partial) *YPL034W* ORF, we refer to the full-length protein product as Yeast Frame Shift 1, Yfs1, and the corresponding gene *YFS1*. The first predicted uORF will be referred to as ORF1 and the N-terminally extended mORF of *YPL034W* will be designated ORF2.

**Figure 3:**
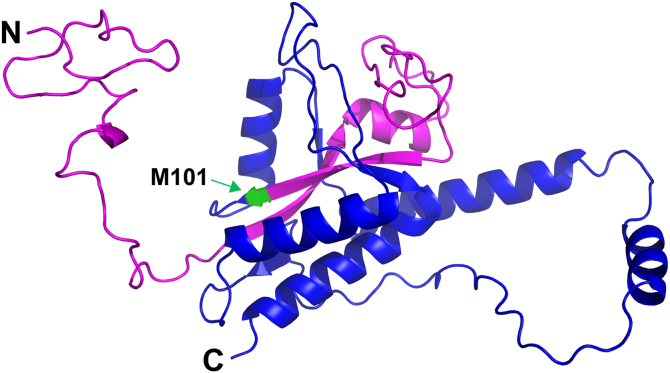
**Alphafold 3 predicted protein structure of Yfs1**. The Alphafold 3 predicted structure of full-length Yfs1 is displayed using PyMol. The previously annotated *YPL034W* residues are colored *blue* with the annotated start codon (M101 in Yfs1) in *green*; the predicted N-terminal extension (residues 1–100) are in *magenta*.

To assess whether the predicted N-terminal extension in Yfs1 contributes to the structure of the full-length protein, we used Alphafold 3 (29) to predict the structure of Yfs1. As shown in Figure 3, the first 47 residues of the 100-residue N-terminal extension in Yfs1 are predicted to be unstructured. The remaining portion of the extension (residues 48–100) are predicted to form two antiparallel β-strands with an intervening loop containing a short α-helix and unstructured region. Notably, this structured portion of the Yfs1 N-terminal extension is predicted to be nestled within the C-terminal portion of the protein. Two short β-strands from the C-terminal portion are predicted to interact with the N-terminal β-strands to form a modest β-sheet structure surrounded by α-helices. As Alphafold predicts a similar structure for the C-terminal half of Yfs1 with and without the N-terminus (see predicted structure of the annotated *YPL034w* ORF protein on the SGD database [https://www.yeastgenome.org/]), it is unclear how significantly the N-terminus contributes to the Yfs1 protein structure. However, the amino acid sequence conservation and Alphafold prediction of secondary and tertiary structures supports the idea that the N-terminal extension is part of the native Yfs1 protein.

### Natural history of the frameshift site

To determine how widespread frameshifting in *YFS1* might be among yeast species, an additional 73 sequences of *YFS1* homologs from the yeast order *Saccharomycetales* were retrieved, for a total of 81 homologs (see Experimental procedures). The sequences were manually examined for their potential or requirement for +1 frameshifting, conceptually translated, and the predicted peptide sequences subjected to additional analysis. Of the 81 homologs, 52 require +1 frameshifting for expression of the full-length Yfs1 protein (including the N-terminal extension), while 29 do not. ClustalX was used to align the predicted amino acid sequences of all 81 proteins and to generate a phylogenetic tree based on this alignment. The alignment (Fig. 2*B*) showed that even though the peptide sequence was diverging at a relatively high rate, 9 of the 96 amino acid residues in the predicted N-terminal extension of ORF2 are identical in at least 90% of the species examined, a level of amino acid conservation higher than the rate of conservation (5 out of 100) in the 100 residues downstream of the methionine corresponding to the annotated start codon of *YPL034W* (amino acid 101 in the alignment, marked with a black arrow in Fig. 2, *A* and *B*), presumably because they are crucial for the function of the protein.

Alignment of the nucleotide sequences surrounding the putative frameshift site from the 52 homologs that require +1 frameshifting showed near-perfect conservation of the CUU-AGG-C heptanucleotide sequence (Fig. 2*D*). In three related species, the heptanucleotide sequence was CUU-CGG-C instead. In addition to the heptanucleotide shift site, several additional nucleotides were highly conserved—a “UG” dinucleotide before the shift site and a “UNAC” motif located downstream. Further analysis of the amino acid sequence conservation revealed that the Cys specified by codon 3 of *S. cerevisiae YFS1* (encoded by UGY) is absolutely conserved in all 81 sequences examined, including 29 that do not require frameshifting, and is therefore likely essential for the function of the protein. This indicates that the UG dinucleotide before the frameshift site is likely conserved because of the importance of the amino acid it encodes rather than its role in frameshifting. The same is probably true for the Thr (ACY) codon at position 7 of the protein that includes the AC dinucleotide of the UNAC motif. Outside the putative heptanucleotide sequence, the only other conserved residue is the “U” of the UNAC motif located two nucleotides downstream of the putative heptanucleotide shift site. As this U is the second base in a variety of triplets encoding multiple different amino acids in the 52 frameshift sequences, its conservation is likely for reasons other than its coding potential and thus could have a role in frameshifting.

**Figure 4:**
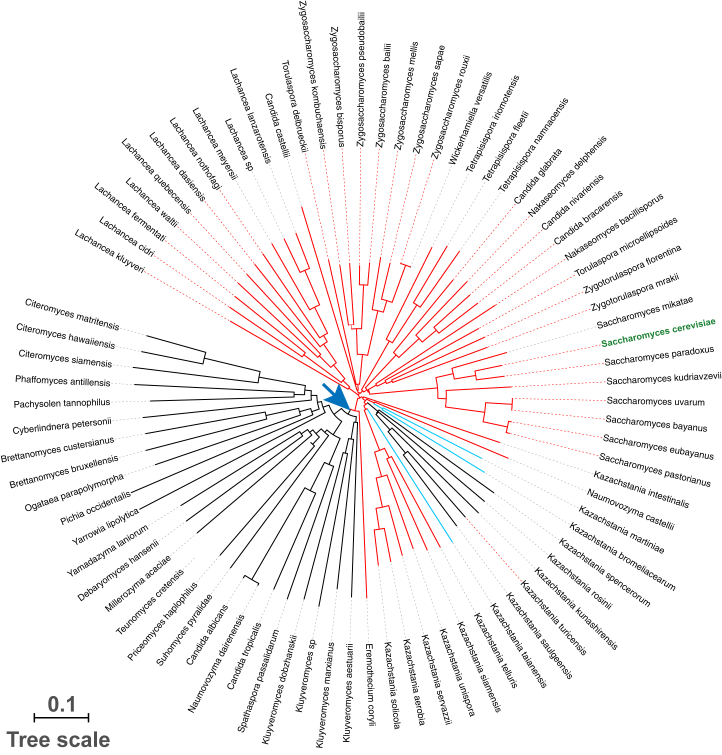
**The +1 frameshifting of *YFS1* is ancient**. Unrooted phylogenetic tree of 81 homologs of Yfs1/YPL034W based on their peptide sequence (after simulated +1 frameshifting) and using ClustalX. Species names are indicated on the periphery. *S. cerevisiae* is in green. The branches of homologs that require +1 frameshifting and have the inferred shift site CUU-AGG-C are in *red*. The branches of homologs with the variant shift site CUU-CGG-C are in *blue*. The branches of homologs not requiring frameshifting are in black. An inferred point of frame shift site emergence is indicated by a *blue arrow*. The absence of frameshifting in the *Kazachstania* species following its emergence in an earlier branch suggests a secondary loss of frameshifting in these species. The scale bar in the lower left measures the number of amino acid substitutions per site (accounting for multiple substitutions at the same site).

The phylogenetic tree of the 81 *YFS1* homologs matched the known phylogenetic relationship (https://www.ncbi.nlm.nih.gov/Taxonomy/) of the corresponding yeast species (Fig. 4). All species that branch prior to the split between the family *Saccharomycetaceae* and the other members of the order *Saccharomycetales* lack a requirement for frameshifting. Members of the genus *Kluyveromyces,* which branched off early during the diversification of *Saccharomycetaceae,* also do not require +1 frameshifting for expression of *YFS1*. This places the emergence of +1 frameshifting in *YFS1* between 100 and 150 million years ago (30, 31, 32). Five species in the *Kazachstania* genus appear to have sustained secondary loss of +1 frameshifting. In addition, three other species in the same genus have evolved a variant of the shift site, CUU-CGG-C, in which the first nucleotide of the triplet occupying the A site in the zero frame is changed from A to C. Of note, the converse change of the Ty1 shift site sequence from the wild type CUU-AGG-C to CUU-CGG-C in *S. cerevisiae* severely reduces the level of +1 frameshifting but does not eliminate it completely (14).

### Ribosome profiling data provide direct support for +1 frameshifting in *YFS1*

**Figure 5:**
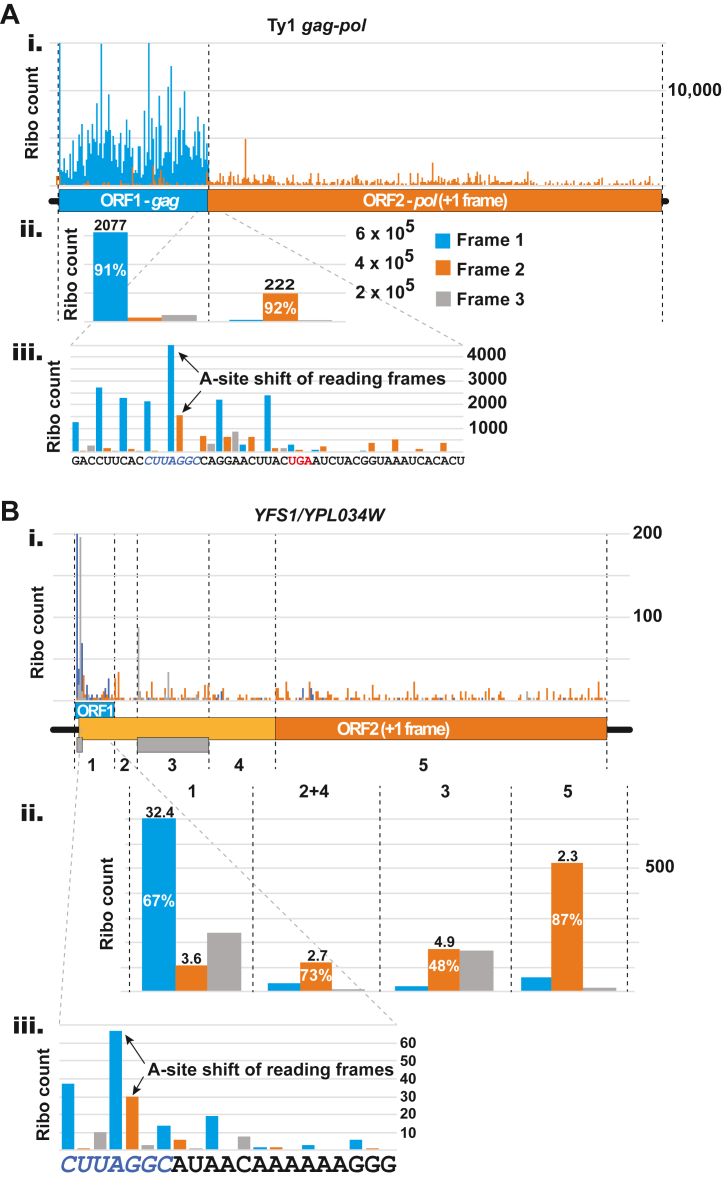
**Ribosome profiling data support +1 frameshifting on the CUU-AGG-C sequence of *YFS1***. Ribosome profiling data from selected publicly available studies with high quality framing information (see Experimental procedures for criteria used, methods of analysis, and sources of the sequencing data) were analyzed for evidence of frameshifting on the CUU-AGG-C sequence of Ty1 *gag-pol* and *YFS1*. Reads (28 nt only) of protected fragments were aligned at the 5′ ends with 15 nt offset to the ribosomal A site. Fragments in blue align to nucleotide “1” of triplets in the “0” reading frame; fragments in orange align to nucleotide “2” of the “0” reading frame (and nucleotide “1” of triplets in the “+1” reading frame); and fragments in *grey* align to nucleotide “3” of the “0” reading frame. (*A*) Ribosome protected fragment alignment to the mRNA of Ty1 *gag-pol*. The regions of ORF1 (*gag*) and ORF2 (*pol*) are delimited by *dashed lines*. *Top panel* (i), global coverage. *Middle panel* (ii), the reads of ORF1 and ORF2 were added and tabulated by sub-codon position. The percent of total reads for the predominant frame are displayed on the columns; numbers above columns are the average reads for that nucleotide position per codon. *Bottom panel* (iii), a blow-up version of the shift site from panel ii. Paused ribosomes with either “AGG” (before shift) or “GGC” (after +1 shift) in the A site are indicated by *arrows*. The corresponding nucleotide sequence of the mRNA is shown at the bottom with the shift site CUU-AGG-C in *blue* and in *italics*, and the UGA stop codon of ORF1 in *red*. (*B*) Ribosome protected fragment alignment to the mRNA of *YFS1* (*YPL034W*). *Top panel* (i), raw read counts are shown above a schematic of the mRNA features. ORF1 is in *blue*; the previously annotated portion of ORF2 (*i.e. YPL034W*) is in *dark orange*, and the 5′ extension of ORF2 is in *light orange*; two short internal ORFs (iORFs) in the third frame, which exhibit evidence of translation, are shown as *gray rectangles*. For analysis purposes, the mRNA is subdivided into different regions numbered 1 to 5 and the boundaries are shown as *dashed lines*. Region “1” = ORF1; regions 2 and 4 = sections of ORF2 that are currently not annotated as coding and do not overlap with iORF2; region 3 = iORF2; region 5 = currently annotated *YPL034W* mORF. *Middle panel* (ii), the reads aligning to the numbered regions are added and tabulated by sub-codon position. Numbers displayed in and on top of the columns represent the same as in (*A*). *Bottom panel* (iii), a blow-up version of the shift site with analysis as in *panel* ii. Paused ribosomes with either “AGG” (before shift) or “GGC” (after +1 shift) in the *A* site are indicated by *arrows*. The corresponding nucleotide sequence of the mRNA is shown at the *bottom* with the shift site CUU-AGG-C in *blue* and *italics*.

Ribosome profiling has the potential to produce exceptional reading frame phase information, especially if analysis is restricted to protected fragments with a certain fixed length and direction of offset. In some ribosome profiling studies of yeast greater than 90% of the 28 nt long fragments show the same 15 nt distance from the 5′ end of the footprint to the first nucleotide of the A-site codon within the ribosome. We analyzed data from several previously published studies that match these criteria and have high ribosomal coverage (see Experimental procedures). These data were used to interrogate the evidence for +1 frameshifting in ORF1 of *YFS1*. To validate the approach, we first examined the evidence for the well-studied example of +1 frameshifting during translation of the *gag-pol* gene of yeast Ty1, which utilizes the same frameshift site, CUU-AGG-C, as the putative site in *YFS1*. In total, more than 1.3 million ribosome footprints from seven studies (see Experimental procedures) were aligned to the CDS of the Ty1 *gag-pol* mRNA. Plotting the reads corresponding to each frame in different colors, zero frame in blue and +1 frame in orange, a sharp change in frame signal was seen at the point of the known frameshifting site (Fig. 5*A* *i*). Before the frameshift site 91% of reads aligned at their 5′ end to the arbitrarily defined frame 1, corresponding to frame 0 of *gag*, but after that point 92% of reads aligned to frame 2, corresponding to frame +1 relative to *gag* and frame zero relative to *pol*. Dividing the riboseq coverage of protected fragments (normalized to total number of codons) coming from frame zero across the entire *pol* ORF (orange in Fig. 5*A* *i-ii*) by those coming from the zero and +1 frames of the entire *gag* ORF (blue in Fig. 5*A* *i-ii*) allowed us to estimate the frequency of frameshifting in *gag-pol* as 10.3% (see Experimental Procedures for details), which is close to the 12% Ty1 frameshifting measured with reporters (16). Notably, a higher frameshifting efficiency of 20% was observed using a different reporter system (15), and even higher levels of frameshifting, 40%, were observed using reporters containing the minimal sequence including only the heptanucleotide CUU-AGG-C (14). The high coverage of ribosome protected fragments for the Ty1 mRNA, with average reads of over 2000 per codon for ORF1, allowed for near nucleotide resolution of translating ribosome positions on this mRNA. As a result, the switch of reading frames was visualized directly (Fig. 5*A* *iii*), where a fraction of ribosomes appear to be moving from the “AGG” (Arg) codon in the zero frame (blue) to the “GGC” (Gly) codon in the +1 frame (orange), with the ribosomes that failed to frameshift continuing in the zero frame (blue) to the stop codon (Fig. 5*A* *iii*).

The same approach used to validate the technique on Ty1 *gag-pol* frameshifting was applied to investigate the putative frameshifting of *YFS1* (Fig. 5*B*). Expression levels of *YFS1* are almost three orders of magnitude lower than those for Ty1 *gag-pol*, making interpretation of the results more challenging. The analysis is also complicated by the presence of two apparently translated internal ORFs encoded in different reading frames within ORF1 or ORF2 (iORFs, grey boxes in Fig. 5*B* *i*). Despite these complications, the data show that 67% of footprints coming from ORF1 of *YFS1* (region 1 of Fig. 5*B* *i-ii*) aligned to frame 1 (frame 0 of ORF1). Seventy-three percent of fragments aligning to the region of ORF2 that extends 5′ of the annotated AUG start, and does not overlap with the second iORF (Regions 2 and 4 of Fig. 5*B* *i-ii*), aligned to frame 2 (frame 0 of ORF2), similar to the 87% of fragments that aligned to frame 2 (frame 0 of ORF2) of the previously annotated section of *YFS1* (region 5 of Fig, 5*B* *i-ii*). The profiling data also supported translation of the two iORFs, both in frame 3. The first iORF is wholly contained within ORF1 (originally predicted to be a uORF), and the second iORF lies within ORF2 of *YFS1* and begins 30 nt downstream of the ORF1 stop codon. Similar to Ty1, and despite the lower coverage, the change of frame in the A-site codon of ribosomes translating *YFS1* was visualized by the aggregate 80S ribosome profiling data (Fig. 5*B*). By dividing the density of footprints coming from frame zero of ORF2 by the density of footprints coming from the sum of frame zero and frame +1 of ORF1 we estimated the rate of +1 frameshifting as 19.3%. Because of the uneven and relatively limited ribosome footprint coverage of both ORF1 and ORF2, the accuracy of this estimate is, admittedly, limited. As noted for the Ty1 frameshift site, the switch of reading frames on *YFS1* could be visualized directly. A fraction of ribosomes appear to be moving from the “AGG” (Arg) codon in the zero frame (blue) to “GGC” (Gly) codon in the +1 frame (orange) (Fig. 5*B* *iii*).

### Luciferase reporters demonstrate efficient +1 frameshifting on *YFS1*

**Figure 6:**
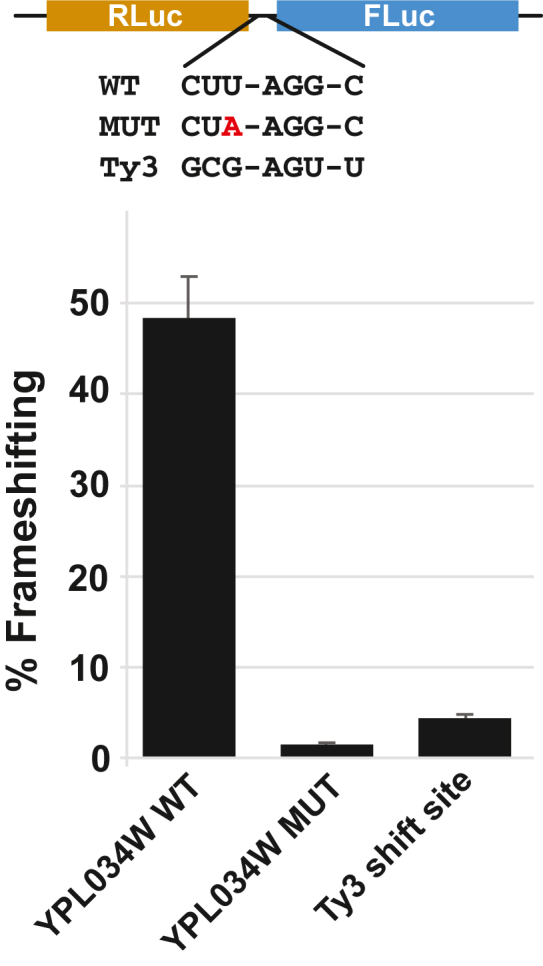
**A *YFS1* frameshift cassette supports efficient +1 frameshifting in *S. cerevisiae*.***S. cerevisiae* cells, strain BY4741, were transformed with dual-luciferase reporters containing either the wild-type (WT) *YFS1* frameshift cassette (60 nt surrounding the putative shift site), a mutant (MUT) cassette in which the *YFS1* shift site is changed from CUU-AGG-C to CUA-AGG-C, or the Ty3 frameshift sequence. In all cases the ratio of firefly to *Renilla* luciferase activity is compared to values obtained from in-frame control constructs, and the results are plotted as percent frameshifting. ∗∗*p* < 0.01 (Student’s two-tailed *t* test; n = 3).

To provide additional evidence that *YFS1* mRNA can support ribosome frameshifting *in vivo*, we inserted a 60-nt sequence spanning the putative frameshift site of *YFS1* and flanking regions, spanning 7 nt upstream to 46 nt downstream of the 7-nt shift sequence, between the *Renilla* and firefly luciferase coding regions in the dual-luciferase reporter pJD375. Control reporters in which the shift site was mutated from CUU-AGG-C to CUA-AGG-C to prevent frameshifting or in which the wild-type Ty3 frameshift site was inserted between *Renilla* and firefly were also generated. The plasmids were introduced into the wild-type *S. cerevisiae* strain BY4741, and the luciferase activity was assayed. When compared to their respective in-frame controls in which *Renilla* and firefly luciferases are encoded in the same reading frame due to single-nucleotide deletions in the *YFS1* or Ty3 frameshift sequences, the *YFS1* frameshift site yielded 48% frameshifting, comparable to the level of frameshifting observed with the isolated Ty1 frameshift sequence (14). In contrast, the mutation disrupting the *YFS1* frameshift sequence reduced frameshifting to 1.5%. The Ty3 frameshift element supported 4.5% frameshifting (Fig. 6), lower but within range of the frameshifting reported previously with a longer frameshift cassette and a different reporter system (20).

### Western analysis of C-terminally tagged YPL034W is consistent with +1 frameshifting and exclusive initiation at the AUG of ORF1

**Figure 7:**
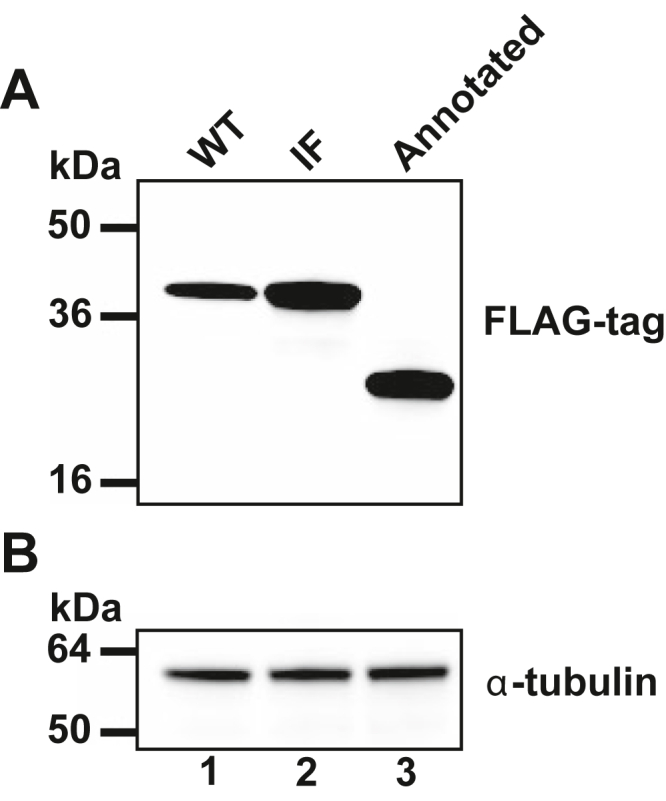
**Immunoblot detection of full-length frameshifted Yfs1 protein.** Whole-cell extracts from yeast strain F353 expressing C-terminally FLAG-tagged *YPL034W* from a galactose-inducible promoter were subjected to immunoblot analysis using (*A*) Anti-FLAG-tag or (*B*) anti-α-tubulin antibody. Lane 1, cells expressing the full length wild-type (WT) *YPL034W/YFS1* sequence; lane 2, cells expressing the full length in-frame (IF, fusing ORF1 and ORF2) *YPL034W/YFS1* sequence; lane 3, cells expressing a truncated *YPL034W/YFS1* sequence starting 6 nucleotides 5′ of the previously annotated AUG start codon.

To investigate whether the YPL034W/Yfs1 protein in yeast initiates at the annotated AUG codon of YPL034w (Met101 in Yfs1) or at the out-of-frame upstream AUG codon (Met1 in Yfs1), C-terminal FLAG-tagged versions of the YPL034W ORF were expressed in yeast under the control of the galactose-inducible *GAL1* promoter in the vector pYES2. The first construct expressed the wild-type sequence of YPL034W starting at the authentic 5′ end of the mRNA, as obtained from the cap analysis gene expression (CAGE) data in the YeasTSS database (33). The second construct was derived from the first construct by deleting a single nucleotide in ORF1, the “A” of the CUU-AGG-C shift site, so that ORF1 and ORF2 are fused in-frame and the YPL034W/Yfs1 protein can be produced without frameshifting. In the third construct, the 5′ end of the *YPL034W* mRNA was truncated by deleting all sequences starting 6 nt upstream of the annotated AUG of *YPL034W*, such that only the annotated YPL034W protein will be expressed. The three constructs were introduced into the wild-type yeast strain F353 and, following growth in galactose media, cell lysates were analyzed by immunoblotting with anti-FLAG-tag antibodies (Fig. 7). Whereas a 25-kDa protein (slightly larger than the expected 22-kDa product) was produced in cells containing the third construct designed to express the annotated YPL034W protein (Fig. 7, lane 3), a 40-kDa protein (slightly larger than the expected 34-kDa size) was detected in extracts from cells containing the in-frame control construct 2 that fused ORF1 and ORF2 (Fig. 7, lane 2). In cells containing the first construct and expressing the authentic YPL034W mRNA, a single 40-kDa protein was detected (Fig. 7, lane 1) that precisely co-migrated with the in-frame control protein (lane 2). These data are consistent with translation of the *YPL034W/YFS1* mRNA producing a single protein that initiates at the AUG of ORF1 (Met1 in Yfs1) and fuses ORF1 and ORF2 *via* +1 frameshifting. Importantly, no 25-kDa protein corresponding to the annotated YPL034W protein (Fig. 7, lane 3) was detected in cells expressing the wild-type *YPL034W/YFS1* construct (lane 1), indicating that the YPL034W/Yfs1 protein is exclusively synthesized by ribosomal frameshifting.

## Discussion

Programmed frameshifting is a well-recognized means of translational regulation of gene expression (9, 10). While occurring frequently in viruses and retrotransposons (9, 10), the number of chromosomal genes whose expression is known to require programmed frameshifting is limited (9). Here we describe the identification of only the fourth example of programmed frameshifting in a chromosomally encoded gene in *S. cerevisiae,* which we call *YFS1*, encompassing the previously identified ORF *YPL034W*.

The hypothesis that *YFS1* requires +1 frameshifting for expression is supported by several lines of evidence. The amino acid conservation for the *YPL034W* ORF2 extends 300 nt upstream of its annotated AUG start codon, but this conserved N-terminal extension lacks its own in-frame initiation codon. An upstream ORF1 is present in all homologs of *YPL034W* from the genus *Saccharomyces,* is always present in the −1 frame relative to *YPL034W* ORF2 and overlaps the conserved N-terminal extension of ORF2. The upstream ORF1 contains the conserved sequence CUU-AGG-C that is known to be sufficient to direct very efficient +1 frameshifting in *S. cerevisiae* and serves as the +1 frameshift site in the *gag-pol* gene of the retrotransposon Ty1 and the chromosomal *ABP140* gene in *S. cerevisiae* and related organisms (31). Aggregate 80S ribosome profiling data (22) show continuous uninterrupted ribosome occupancy in the gap between ORF1 and the annotated start codon of *YPL034W* ORF2. Reading frame analysis indicates that translation of this intervening region is in the +1 frame relative to ORF1 and hence in the same frame as *YPL034W* ORF2. The same data also suggest that the change in reading frame begins at the exact position of the CUU-AGG-C sequence, with the ribosome A site switching from the AGG Arg codon in the 0 frame to the GGC Gly codon in the +1 frame.

Farabaugh *et al.* (31) showed that there is a strong correlation between the presence and the absence of certain tRNA species capable of decoding the CUU Leu codon of the frameshift site and the presence or absence of +1 frameshifting in the *ABP140* and *EST3* genes. Specifically, tRNA^L^_I_^e^_A_^u^_G_ is almost always absent in organisms with +1 frameshifting in *ABP140* and *EST3*, but present in organisms that express these genes without frameshifting, whereas tRNA^L^_U_^e^_A_^u^_G_ is abundant in organisms with +1 frameshifting in *ABP140* and *EST3* and scarce in organisms without +1 frameshifting in these genes. Ivanov *et al.* (34), likewise, found a correlation between the identity of the P-site codon during yeast antizyme +1 frameshifting and the absence of certain tRNA species in specific branches of yeast evolution. This is consistent with the idea that near-cognate tRNA in the P site, in combination with a scarce A-site codon, is a driving force behind efficient +1 frameshifting, as first proposed by Hansen *et al.* (12). The tRNA^L^_U_^e^_A_^u^_G_ that most frequently decodes the CUU Leu codon in *S. cerevisiae* is unusual in that the uridine in the wobble position of the anticodon is not modified, unlike in more distant yeast relatives where modification of the U restricts it to reading A or both A and G. As a result, tRNA^L^_U_^e^_A_^u^_G_ can decode all six Leu codons in *S. cerevisiae* (35, 36). Relatively weak pairing of U•U in the wobble position appears to make tRNA^L^_U_^e^_A_^u^_G_ unusually prone to frameshifting when a CUU codon is present in the P site. Work by Vimaladithan and Farabaugh (37) showed that 11 P-site codons, including CUU, can support appreciable levels of +1 frameshifting. Overexpression of the cognate tRNA for seven of these codons reduced frameshifting, suggesting that frameshifting occurs when the P-site codon is occupied by near-cognate tRNA (38).

The emergence of +1 frameshifting in *YFS1* during evolution matches almost perfectly the emergence of the related +1 frameshifting sites in the yeast genes *ABP140* and *EST3*. In those cases as well, no species prior to the evolutionary divergence between *Kluyveromyces* and other *Saccharomycetaceae* shows a requirement for +1 frameshifting for expressing *ABP140* and *EST3* (31). This evolutionary pattern is in agreement with the phylogenetic distribution of tRNA_Leu_ isoacceptors and frameshift sites of ABP140 and EST3 in fungal genomes, as determined previously (31), which, as outlined above, appears to drive the shiftiness of CUU-A in *S. cerevisiae*, thus emphasizing the intimate link between the two events.

The *YPL034W/YFS1* gene is non-essential in *S. cerevisiae,* and no functions have been assigned to the protein. The closest homolog of Yfs1 that can be identified in BLAST searches is the *Saccharomyces pombe* protein mug113 (SPAC3F10.05c). The *mug113* gene was identified in a screen for genes whose expression was upregulated during meiosis (39, 40); however, no meiotic phenotypes were observed upon deleting *mug113* in *S. pombe*. Like mug113, the central domain of the YPL034w/Yfs1 protein shows sequence similarity to the GIY-YIG nuclease family. These nucleases are involved in many cellular processes related to DNA repair and recombination, including transposon mobility (41). Consistent with a function related to DNA repair, synthetic genetic array studies (42) revealed that cells lacking *YPL034W* showed similar genetic interactions as cells lacking the Holliday junction resolvase *YEN1*, with both genes showing strong synthetic growth defects when deleted in combination with *POL12* encoding the B subunit of the DNA polymerase α-primase complex. As *POL12* is required for mitotic and premeiotic DNA replication, this genetic linkage of *YPL034W* to *POL12* might also relate to the meiotic function of the *mug113* gene in *S. pombe*. As *YPL034W* also shows strong genetic interactions with loss of the RNA polymerase III subunit *TFC6*, which does not show genetic interactions with *YEN1*, additional studies will be needed to definitively reveal the function of the Yfs1 protein and to determine whether it possesses nuclease activity or functions during meiosis in budding yeast. It will also be of interest to learn whether the utilization of +1 frameshifting for the expression of *YFS1* serves a regulatory purpose.

## Experimental procedures

### Sequence compilation and analysis

Sequences were obtained from GenBank using the BLAST algorithm (43) and applying a strategy described previously (44) by querying the Nucleotide collection (nr/nt), the RefSeq Genome Database (refseq_genomes), and Whole-genome shotgun contigs (wgs) databases. Sequences were aligned using ClustalX (45). The phylogenetic figure was drawn using the browser-based tool iTOL (https://itol.embl.de/) (46). Logogram figures were generated using the WebLogo browser tool (https://weblogo.berkeley.edu/logo.cgi) (47).

### Analysis of ribosome profiling data

The ribosome profiling data were analyzed using the web browser-based RiboGalaxy tools (48). Briefly, reads from wild-type *S. cerevisiae* ribosome profiling data from Guydosh and Green 2014 (49), Guydosh and Green 2017 (50), Young *et al.* 2015 (51), Young *et al.* 2018 (52), Young *et al.* 2021 (53), Gaikwad *et al.* 2021 (54), and Santos *et al.* 2019 (55) were aligned to the specified mRNA transcript (Ty1 *gag-pol* or *YFS1*) using Bowtie. Up to 2 valid alignments were allowed per read. The minimum seed length was set at 25 nt. CSV files were generated with the reads coming from fragments of 28 nt mapped to each gene, with the 5′ end offset from the A site by 15 nt. These files, with the fragment counts per nucleotide position, were used to calculate coverage for each reading frame and to generate figures for that coverage.

### Calculating frameshifting from ribosome profiling data

To calculate inferred frameshifting from aggregate ribosome profiling data, ribosome reads mapping to ORF2 in the +1 frame after the frameshift site were normalized to reads mapping to ORF1 in the 0 and + 1 frames. Ribosomes translating ORF1 will be in the 0 frame before the frameshift site and in the 0 and + 1 frame after the frameshift site. To account for all ribosomes that initiated translation on ORF1, all reads in frames 0 and + 1 mapping between the ORF1 start and stop codons were assigned to ORF1. By contrast, to calculate ribosome density on ORF2 only reads in the +1 frame (relative to ORF1) between the shift site and the ORF2 stop codon were considered. Finally, percent frameshift efficiency was calculated by dividing the density of reads in ORF2 by the density of reads in ORF1 and then multiplying by 100.

### Plasmid construction

The plasmids listed in Table S3 were constructed by annealing oligos listed in Table S2 bearing the respected frameshift sequences with *Sal*I/*Bam*HI overhangs and then cloning into pJD375 (16). The FLAG-tag *YPL034w* expressing plasmids were constructed commercially (LifeSct LLC) as follows: a DNA fragment containing the *YPL034W* sequence starting 333-nts upstream of the annotated AUG (Met101 in Yfs1) and extending to the last sense codon the annotated ORF, followed by DNA encoding (Ala-Gly)_2_-2xFLAG-TAA-ACT was synthesized and inserted between the *Bam*HI and *Xba*I restriction sites of the vector pYES2 (ThermoFisher), generating the wild type plasmid pC7751. An identical DNA fragment but lacking the “A” nucleotide of the CUU-AGG-C shift site was likewise inserted into pYES2 to generate the in-frame control plasmid pC7752. The annotated YPL034w plasmid pC7753 matches the sequence of pC7751 except the YPL034w sequence starts just 6 nucleotides upstream of the annotated AUG (Met101 in Yfs1) and was constructed analogously.

#### Dual luciferase assay

Transformants of yeast strain BY4741 (*MATa his3Δ1 leu2Δ0 met15Δ0 ura3Δ0*) (Research Genetics) carrying dual-luciferase reporter plasmids (Table S1) were grown in synthetic complete medium lacking uracil (SC-ura) to OD_600_ = 0.8. Next, the cells in 1.5 ml of the exponentially growing culture were harvested and then lysed with glass beads in 400-μl ice-cold lysis buffer (1 × PBS containing two Complete EDTA-free Protease Inhibitor Cocktail Tablets (Roche)/50 ml). *Renilla* and firefly luciferase activities in 5 μl 1:10 diluted lysate were measured using the Dual-Luciferase Reporter Assay System (Promega) as described by Dyer *et al.* (56) with a small modification in the Stop and Glo substrate (the homemade Stop and Glo buffer was supplemented with 5% commercial Stop and Glo buffer purchased from Promega). The relative light units were measured using a CentroXS3 LB960 microplate luminometer fitted with two injectors (Berthold Technologies). For the YPL034W and Ty3 dual luciferase reporters, *Renilla* luciferase activity from the downstream cistron was normalized to firefly luciferase activity from the upstream cistron, and the values obtained with the test reporters were compared to “in-frame” controls to calculate percent frameshifting. In contrast, in case of the Ty1 dual luciferase reporters, firefly luciferase activity from the downstream cistron was normalized to *Renilla* luciferase activity from the upstream cistron and the values obtained with the test reporters were compared to the in-frame empty vector pJD375 control to calculate percent frameshifting.

#### Quantification and statistical analysis

Luciferase assay data are presented as the mean and standard deviation from biological replicates indicated in the figure legends. The statistical significance between groups and comparisons were made using Student’s two-tailed t tests, using the formula embedded in Microsoft Excel. *p* values less than 0.05 were considered significant.

#### Expression and immunoblotting of C-terminally FLAG-tagged YPL034W

Transformants of yeast strain F353 (MATα *ura3-52 trp1 leu2-Δ1 his3-Δ200 pep4::HIS3 prb1-Δ1.6R can1 GAL*^*+*^) were grown in synthetic complete (SC) dextrose medium containing all amino acids and lacking uracil to OD_600_ = ∼2 and collected by centrifugation. Cells were washed with 20% trichloroacetic acid (TCA), resuspended in 200-μl 20% TCA, mixed with the same volume of glass beads, and then vigorously mixed on a vortex for 2 min. Following centrifugation at 3000 rpm at room temperature (RT) for 10 min, the protein pellet was resuspended in 100-μl 2x Laemmli sample buffer (Bio-Rad #1610737) and neutralized by adding 100-μl 1 M Tris-base. Following heating at 80 °C for 10 min, samples were separated by SDS-polyacrylamide gel electrophoresis and then subjected to immunoblot analysis with rabbit anti-FLAG antibodies (Abcam EPR20018–251). Blots were stripped and re-probed with rabbit anti-α-Tubulin antibodies (Abcam EPR13799). Immune complexes were detected using horseradish peroxidase conjugated anti-rabbit IgG (GE Healthcare NA9340V) developed with Radiance Q chemiluminescent substrate (Azure BioSystems AC2101) and chemiluminescence was imaged using an Azure BioSystems 280 machine.

## Data availability

All data are contained within the manuscript.

## Supporting information

This article contains supporting information (58).

## Conflict of interest

The authors declare that they have no conflicts of interest with the contents of this article.
