# Supplementary material for: Conserved +1 translational frameshifting in the Saccharomyces cerevisiae gene encoding YPL034W
Source: J Biol Chem. 2025 Nov 4;301(12):110891. doi: 10.1016/j.jbc.2025.110891 (PMC12719033; doi:10.1016/j.jbc.2025.110891)
Supplement: Supplemental Tables [file mmc1.docx]

**Table S1. Plasmids used in this study**

| Plasmid | Description | Source/Reference |
| --- | --- | --- |
| YPL034W_WT | YPL034W frameshift site in pJD375 | This study |
| YPL034W_MUT | YPL034W mutated frameshift site in pJD375 | This study |
| YPL034W_IF | YPL034W inframe frameshift site in pJD375 | This study |
| Ty3_WT | Ty3 frameshift site in pJD375 | This study |
| Ty3_IF | Ty3 inframe frameshift site in pJD375 | This study |
| pJD204.+1 | Ty1 frameshift site in pJD375 | (57) |
| pJD375 | Dual-luciferase vector | (16) |
| pC7751 | Full-length YPL034W-2xFLAG in pYES2 | This study |
| pC7752 | Full-length inframe YPL034W-2xFLAG in pYES2 | This study |
| pC7753 | Annotated YPL034W-2xFLAG in pYES2 | This study |
| pYES2 | Yeast galactose-inducible expression vector | ThermoFisher |

**Table S2. Oligonucleotides used in this study**

| Primer name | Sequence |
| --- | --- |
| YPL034W_WT/S | TCGACGAGATGTCTTAGGCATAACAAAAAAGGGAGAGCGGTGCAAGATAAACATACCGTCGGATAAAG |
| YPL034W_WT/A | GATCCTTTATCCGACGGTATGTTTATCTTGCACCGCTCTCCCTTTTTTGTTATGCCTAAGACATCTCG |
| YPL034W_MUT/S | TCGACGAGATGTCTAAGGCATAACAAAAAAGGGAGAGCGGTGCAAGATAAACATACCGTCGGATAAAG |
| YPL034W_MUT/A | GATCCTTTATCCGACGGTATGTTTATCTTGCACCGCTCTCCCTTTTTTGTTATGCCTTAGACATCTCG |
| YPL034W_IF/S | TCGACGAGATGTCTTGGCATAACAAAAAAGGGAGAGCGGTGCAAGATAAACATACCGTCGGATAAAG |
| YPL034W_IF/A | GATCCTTTATCCGACGGTATGTTTATCTTGCACCGCTCTCCCTTTTTTGTTATGCCAAGACATCTCG |
| TY3_WT/S | TCGACGCGTAAGGCGAGTTCTAACCGATCTTGAAG |
| TY3_WT/A | GATCCTTCAAGATCGGTTAGAACTCGCCTTACGCG |
| TY3_IF/S | TCGACGCGTAAGGCGGTTCTAACCGATCTTGAAG |
| TY3_IF/A | GATCCTTCAAGATCGGTTAGAACCGCCTTACGCG |

**Table S3. Plasmids made for this study**

| Construct name | S primer | AS primer |
| --- | --- | --- |
| YPL034W_WT | YPL034W_WT/S | YPL034W_WT/A |
| YPL034W_MUT | YPL034W_MUT/S | YPL034W_MUT/A |
| YPL034W_IF | YPL034W_IF/S | YPL034W_IF/A |
| TY3_WT | TY3_WT/S | TY3_WT/A |
| TY3_IF | TY3_IF/S | TY3_IF/A |
